# Feasibility of a new transmural care pathway for advance care planning for older persons: A qualitative study into community care registered nurses’ perspectives

**DOI:** 10.1016/j.ijnsa.2024.100264

**Published:** 2024-11-08

**Authors:** Patricia Jepma, Roel Eijk, Annigje A.E. Bos, Noor Toet, Corine H.M. Latour, Bianca M. Buurman, Marjon van Rijn

**Affiliations:** a Department of Medicine for Older People, Amsterdam UMC, Amsterdam, the Netherlands; b Amsterdam Public Health, Aging & Later Life, Amsterdam, the Netherlands; c Dutch Healthcare Authority (NZa), Utrecht, the Netherlands; d Amsterdam University of Applied Sciences, Research Group Integrated Complex Care, Faculty of Health, Center of Expertise Urban Vitality, Amsterdam, the Netherlands; e Regioplatform, the Netherlands; f Department of Internal Medicine, Section of Geriatric Medicine, Amsterdam UMC, Amsterdam, the Netherlands

**Keywords:** Advance care planning, Feasibility studies, Implementation science, Frail elderly, Nurses, Community health, Palliative care, Qualitative research, Transitional care

## Abstract

**Background:**

Transmural palliative care interventions aim to identify older persons with palliative care needs and timely provide advance care planning, symptom management, and coordination of care. Nurses can have an important role in these interventions; however, their expertise is currently underused. A new transmural care pathway with a central role for the community care registered nurse in advance care planning aims to contribute to the quality of palliative care for older persons.

**Objective:**

To examine the perspectives of community nurses on the feasibility of a new transmural care pathway for advance care planning for older persons.

**Design:**

A qualitative study design using semi-structured interviews.

**Setting(s):**

Interviews were performed with community nurses of three participating homecare organizations in the Netherlands between March and May 2023.

**Participants:**

19 community nurses.

**Methods:**

A topic guide was based on (1) challenges in advance care planning identified from the literature and (2) concepts that are important in assessing the feasibility of complex healthcare interventions provided by the Normalisation Process Theory framework. A combined inductive and deductive thematic analysis was performed.

**Results:**

Four themes were identified: views on the transmural care pathway, community nurses’ needs to fulfil their role, key points regarding implementation, and evaluation of the new practice. In general, community nurses were positive about the feasibility of the new practice as it provided a more structured work process that could facilitate interprofessional collaboration and improve the quality of palliative care. Overall, the feasibility of the new practice, from community nurses perspective, was determined by (1) clear roles and responsibilities in the transmural care pathway, (2) standardized registration of advance care planning, and (3) close involvement of community nurses in the whole implementation process.

**Conclusions:**

We highlighted important factors, from the perspectives of community nurses, that need to be considered in the implementation of a new transmural care pathway for advance care planning. A clear division of roles and responsibilities, standardized registration of advance care planning, and involvement of community nurses during the whole implementation process were mentioned as important enabling factors. This knowledge might contribute to successful implementation of a transmural care pathway that aims to enhance the quality of palliative care for older persons.

**Tweetable abstract:**

Community nurses’ perspectives on the feasibility of a transmural care pathway for advance care planning for older persons.

**What is already known about the topic**

• Transmural palliative care interventions aim to early identify older persons with palliative care needs and to (timely) provide advance care planning, symptom management, and coordination of care.
• A new transmural care pathway with a central role for the community care nurse in advance care planning aims to contribute to the quality of palliative care for older persons.
• Community care nurses perspectives’ on the feasibility of a new developed pathway are essential, as they might provide important input to consider before implementation.

Alt-text: Unlabelled box

**What this paper adds**

• In general, community nurses were positive about the feasibility of the new practice as it provided a more structured work process that could facilitate interprofessional collaboration and improve the quality of palliative care.
• The feasibility of a transmural care pathway, from community nurses’ perspectives, was determined by (1) clear roles and responsibilities, (2) standardized registration of advance care planning, and (3) close involvement of community care nurses during implementation.
• This knowledge might contribute to the implementation of a new transmural care pathway that aims to enhance the quality of palliative care for older persons.

Alt-text: Unlabelled box

## 1 Background

As a result of the aging population and policy measures, more older persons will experience their last phase of life at home. The presence of life-limiting and progressive chronic diseases such as heart failure and chronic obstructive pulmonary disease may indicate a need for palliative care.(Ganz et al., 2021; Jabbarian et al., 2018) Therefore, advance care planning becomes increasingly important for older persons living at home as it enables them to timely define and discuss their goals and preferences for future medical treatment and care with relatives and healthcare professionals.(Rietjens et al., 2017) In addition, advance care planning also includes the appropriate registration in the medical and nursing files, hand-over of preferences to other involved healthcare professionals, and regular evaluations to ensure that registered preferences remain up to date.

Numerous challenges with regards to the initiation, interprofessional collaboration, documentation, and hand-over of advance care planning wishes and preferences have been described in the literature.(Bernard et al., 2020; Glaudemans et al., 2015; Hemsley et al., 2019; Rietjens et al., 2017; Standing et al., 2020; Stewart et al., 2011) These challenges increase the risks of unwanted care transitions in the end of life; e.g., acute hospitalizations and not dying at the preferred place of death. To overcome these challenges, transmural care interventions have been developed. These interventions aim to provide coordinated patient-centred care in close collaboration and joint responsibility between involved general and specialised healthcare professionals.(van der Linden et al., 2001) For transmural palliative care, interventions might consist of the early identification of older persons with palliative care needs, advance care planning, symptom management, and coordination of care between hospital and home.(Flierman et al., 2023) Nurses can have an important role in these interventions; however, optimal interprofessional collaboration in palliative care is still insufficient.(van Doorne et al., 2023).

Currently, a new transmural care pathway to better organize advance care planning for older persons has been developed in a region of the Netherlands.(Regioplatform, 2024) Community care registered nurses (hereafter community nurses) will play an important role in the timely initiation of advance care planning for older persons in close collaboration with the general practitioner or hospital specialist. Furthermore, they will play a central role in care coordination of palliative care interventions. While much research has been done into developing advance care planning initiatives, important factors for implementation of these interventions in community care in the Netherlands have not yet been described well in the literature,(Francke et al., 2020; Wilkin et al., 2024) or the description is specifically focused on the perspectives of physicians.(Glaudemans et al., 2019) Community nurses’ perspectives on the feasibility of the pathway are essential as their expertise and experience might provide important input to consider before implementation. Therefore, we examined the perspectives of community nurses’ on the feasibility of a transmural care pathway for advance care planning for older persons.

## 2 Methods

### 2.1 Design

A qualitative study design was used to examine the perspectives of community nurses on the feasibility of a new transmural care pathway for advance care planning for older persons.

### 2.2 Participants

Community nurses from three different homecare organizations in the region North-Holland North of the Netherlands were included. Both purposive and snowball sampling were used to acquire a strategically chosen sample of community nurses with varying levels of experience and working at different homecare organizations.(Acharya et al., 2013; Palinkas et al., 2015) The first recruitment was performed by the managers of participating homecare organizations, and additional participants were recruited via snowballing until data saturation was reached.

### 2.3 The transmural care pathway for advance care planning

**Fig. 1:**
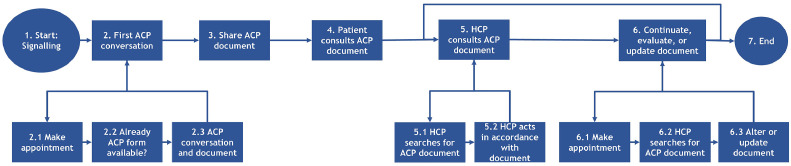
Process workflow of the concept transmural care pathway advance care planning Regioplatform.

Abbreviations: ACP: advance care planning; HCP: healthcare professional

The transmural care pathway was developed in a regional coalition of different care organisations (called Regioplatform), including hospitals, general practitioner groups, homecare organisations, Center of Expertise of the Amsterdam University of Applied Sciences, and the department of medicine for older people of the Amsterdam University Medical Center. The aim of the development and implementation of a new transmural care pathway is to facilitate improvement of transmural and interprofessional advance care planning for older persons in the region North-Holland North in the Netherlands (Regioplatform, 2024). The pathway consists of a uniform work process for all involved healthcare professionals with a clear division of roles and responsibilities (Fig. 1). In addition, a standardized paper-based form has been developed for the documentation of older persons’ advance care planning preferences, which will be made available for all involved healthcare professionals, the client, and relatives. The new form consists of multiple sections in which both physicians and community nurses have a role. Physicians will be responsible for the medical section in which clients’ wishes regarding medical treatment options and limitations are registered (e.g., regarding prescription of antibiotics, resuscitation, hospital admissions, and intensive care admissions). Nurses will be responsible for the care preferences section (e.g., regarding functional decline, existential and spiritual needs and quality of life) [available upon request]. The form was based on existing national and regional formats for advance care planning and on topics that were already present in the different digital systems of participating healthcare organizations. A central regional healthcare platform to digitally register advance care planning preferences is currently being developed and will be pilot-tested at the beginning of 2025.

### 2.4 Data collection

Semi-structured interviews in Dutch were conducted between March and June 2023 at the team office of the home care organisations. A pilot interview was performed by two researchers (PJ and RE). Both were guided by additional training in qualitative research and had previously performed qualitative studies. After the first pilot interview and adjustments in the topic guide, RE performed all following interviews. Neither researcher had any previous relationships with the included participants.

The topic guide was based on the Normalisation Process Theory conceptual framework (May et al., 2022) and findings from the literature search about challenges in the advance care planning process. The Normalisation Process Theory framework provides insight into factors that facilitate or impede the normalization of complex healthcare interventions in practice (May and Finch, 2009). It consists of four fundamental constructs that are essential for normalization of a new practice: coherence, cognitive participation, collective action, and reflexive monitoring (Supplementary Material File 1) (May et al., 2024). Specifically for the evaluation of complex healthcare interventions, the model has been extensively researched, and its constructs have been well-validated (McEvoy et al., 2014).

Prior to the interview, all participants received information regarding the work process in the transmural care pathway, were able to read the new advance care planning documentation form, and see how both would function in practice. All interviews were audio recorded and lasted between 38 and 74 minutes . To guarantee validity of the interview transcripts, a member check was done by sending a one-page summary of the interview to participants (Birt et al., 2016).

### 2.5 Data analysis

All interviews were transcribed ad verbatim in Microsoft Word. Then, analysis was done by means of qualitative data analysis software MAXQDA 2020, using a combination of inductive and deductive thematic analysis. Inductive thematic content analysis allows themes to emerge from the data, while with deductive analysis the data is analysed with preconceived themes based on the Normalization Process Theory framework (Elo and Kyngäs, 2008). Coding was performed in several rounds as an interactive process to strive for theoretical saturation (Corbin and Strauss, 1990). Starting off, open coding (by RE and MvR) was applied to the first three interview transcripts to identify initial concepts. Thereafter, axial coding was done by reorganizing the concepts and clustering the different codes into an initial coding framework. In a final round of selective coding, subcategories and categories were further defined and structured which led to the final coding framework. Lastly, corresponding quotes were selected and translated in English, the research question was answered and findings were compared with the literature.

### 2.6 Ethics approval and informed consent

The Medical Research Ethics Committee of the Amsterdam University Medical Centers approved this study (Reference number 2022.0869). Prior to the interview, participants received oral and written information, and informed consent was obtained.

## 3 Results

In total, 19 community nurses were interviewed. All were female and aged between 22 and 62 years (mean 39.3 years, SD 13.1). Their years of experience as healthcare professionals varied between 1 and 20 years (median 5 years, IQR 3–8). In total, 79 % of the community nurses provided mainly somatic care in their current function (Table 1).

**Table 1:** Participant characteristics.

#	Specific function as community nurse	Age	Years of experience as healthcare professional	Organization
1	Dementia care/case manager dementia	55	5	A
2	Dementia care	25	3	A
3	Dementia care	22	2	A
4	Dementia care	52	2	A
5	Somatic care	46	8	A
6	Somatic care	62	8	A
7	Somatic care	24	3	A
8	Somatic care	31	8	A
9	Somatic care	60	3	B
10	Somatic care in training	51	/	B
11	Somatic care/technical homecare	30	6	B
12	Somatic care	36	9	C
13	Somatic care	47	12	C
14	Somatic care	46	20	A
15	Somatic care	27	5	A
16	Somatic care/ palliative care consultant	33	7	B
17	Somatic care	46	4	B
18	Somatic care	26	1	C
19	Somatic care	28	1	C

Four themes were identified: 1) views on the transmural care pathway, 2) community nurses’ needs to fulfil their role, 3) key points regarding implementation and 4) evaluation of the new practice.

### 3.1 Views on the transmural care pathway

#### 3.1.1 Continuity of care in advance care planning

Most community nurses believed that the transmural care pathway enabled a more structured work process, which could help them to early identify older persons with palliative care needs and, as a result, to initiate timely advance care planning:

*“So you do not have to do everything last minute, when everything is in a very intense phase and where you can't think properly. Sometimes you have to act quickly, so that it is simply clear for the general practitioner, for the community nurse, for the hospital what needs to be done or what a client would like. So I think it is very good if this has already been discussed with the client on time.” [P19]*

Community nurses also mentioned that the pathway and the standardized documentation form could help to create uniformity in the provision of advance care planning by different healthcare professionals; e.g., regarding the topics that will need to be discussed and registered. They indicated that this could prevent multiple advance care planning conversations by different involved healthcare professionals, which might reduce the burden for both clients and involved healthcare professionals.

*“How often do you hear from clients that they have to tell the same story every time? And that it is also interpreted differently. And that nothing more is done with it. And I think that [the standardized registration of advance care planning preferences] makes a difference for the client. And for us too. Because we don't all have to ask out the list again.” [P9]*

Community nurses indicated that the form might also help in case of an acute situation as all involved healthcare professionals could easily access older persons’ care and treatment preferences. They mentioned that the uniform work process could improve the collaboration between all involved healthcare professionals in the region, and subsequently, the quality of advance care planning for older persons.

*“It is more that it can create a certain level of peace and clarity... and that everyone is working on the same goal. So I think it is directly going to yield a lot more for the client.” [P16]*

#### 3.1.2 Engaging clients in advance care planning

Several community nurses highlighted the need to add a client version of the form with easily understandable terminology to engage clients in the advance care planning process, which might help to enhance clients’ empowerment and shared-decision making:

*“I consider client empowerment to be the most important thing. So that the client can fill in things themselves. And that it is less about what we can do and more about what the client do.” [P12]*

Community nurses recommended that the client version of the form should be a physical one that they can provide to clients prior to engaging in advance care planning conversations together as many older persons lack digital skills. Community nurses expected that the discussion of sensitive topics might be easier when clients have the opportunity to prepare themselves beforehand:

*“Where you simply let the client think for themselves... about what their desires are. And not immediately engage in heavy conversation.”[P14]*

Community nurses were generally positive about the structure of the advance care planning documentation form, as it was built holistically and included both psychosocial and medical preferences. They mentioned that engaging clients more in advance care planning, with help of the preparation tool, might contribute to the delivery of personalized care and older persons’ quality of life. As one community nurse explained, the form could help to address what quality of life actually entails for each individual client:

*“I cannot determine what quality of life means to someone. The new document will indicate that, which we can adhere to.” [P17]*

#### 3.1.3 Recommended adaptations to the documentation form

Some community nurses elaborated on changes that should be made to the documentation form before implementation. First, several community nurses indicated that the wording and approach of the form were heavily focused on end-of-life and death. They recommended that the form should be framed and articulated as a method to discuss older persons preferences and wishes with the aim to improve clients’ quality of life. This approach would reduce the taboo surrounding end-of-life discussions and make the form less intimidating.

*“With this title, the client won't understand it at all. So you should actually say, "This is a form where we would like to discuss quality of life with you." Then people would also be more intrigued.” [P6]*

Furthermore, most community nurses also found the form too lengthy for a single advance care planning conversation, which might burden clients. They suggested to divide the advance care planning discussion into multiple sessions. Additionally, they expressed a desire to have flexibility in the order of topics based on the information provided by the client to enhance the effectiveness of the advance care planning process:

*“I would definitely use it, but I would rearrange the order of the document for myself.” [P11]*

### 3.2 Community nurses’ needs to fulfil their role

#### 3.2.1 Clear structure of roles and responsibilities

All community nurses emphasized the importance of a clear structure of roles and responsibilities before implementing the transmural care pathway. They highlighted the need to determine the appropriate timing of initiation, which clients should be included, and which healthcare professionals should be involved.

*“Well, actually, the question that immediately came to my mind was: where does the responsibility for the form lie? Which part belongs to whom? And when is it filled out?”[P8]*

They recommended assigning one coordinating healthcare professional who allocates specific responsibilities for different parts of the form and establishes effective communication among all involved healthcare professionals. Most community nurses believed that this might be their responsibility because of their expertise in care coordination. They expressed their suitability for initiating advance care planning and discussing the psychosocial care preferences because of their extensive knowledge in this area. Community nurses emphasized that their close relationships with clients, built through regular contact and trust, might facilitate their role in (initiating) advance care planning conversations:

*“As community nurses, you have daily contact with the clients at their homes. You are familiar with their situation, and you have the time and the relationship to engage in open conversations. Simply give the client time to think about it, to initiate the process, and let people thinking about these things” [P8]*

Community nurses recommended that the medical care preferences section should be discussed by a general practitioner because of their responsibility for medical care and because they could better explain the potential consequences of clients' preferences regarding future treatments and treatment limitations.

*These sections are better suited for the expertise of a general practitioner who can provide clearer explanations. I can also provide some explanation, but it falls more within the responsibility of the general practitioner.” [P18]*

Several community nurses proposed a working process where they would first engage in discussions with the clients regarding the general and social care preferences sections and then refer them to a general practitioner for the medical section and the registration of treatment limitations.

*“Discussing this form takes time. […] I think the community nurse can make a good start and then tell the client that there are some additional questions that need to be discussed with the general practitioner. So, I ask the client to make an appointment [with the general practitioner] and tell them that I will ask the doctor in four weeks what has been discussed. So you put some pressure behind it.” [P6]*

#### 3.2.2 Clarity on eligible clients

Community nurses recommended to consider carefully which clients receiving home care would be eligible for the new transmural care pathway. Some community nurses noted that differences in client populations might influence the relevance for the new practice and therefore also the implementation. For example, some organizations provided mainly short-term care and experienced a high turnover of clients, while other organizations provided more long-term or palliative care. Community nurses mentioned that this might influence the feasibility of initiating advance care planning:

*“For example, we receive a client for drips after cataract surgery. Then you are involved in care for 3 weeks. As a community nurse, you will do an intake and probably not even an evaluation as it is such a specific defined care need. You also do not create a building of trust.” [P1]*

#### 3.2.3 Knowledge and skills on advance care planning

While discussing the skills and training needed to perform the new practice, community nurses mentioned that advance care planning still gets little attention in nursing education, resulting in relatively low advance care planning awareness and knowledge:

*“Advance care planning, palliative care, and end-of-life care are truly neglected areas. There are many national projects, but the concept of advance care planning is still not well understood in the community.” [P16]*

Many community nurses expressed the need for enhanced skills in conducting advance care planning conversations and to learn more about addressing difficult topics, appropriate conversation skills, and managing emotions. Role playing was frequently suggested as an effective method of training.

In addition, several community nurses believed that additional training was needed to gather additional skills in advance care planning regarding the wishes and preferences of clients and relatives. They expressed the wish to be able to provide explanations on what is actually feasible and having the knowledge to know how to intervene:

*“When you hear those preferences, it's nice that the person who administers the form also has the knowledge to say, ‘Hey, that can be done through the municipality or in this way, another type of support or volunteers’ so that immediate actions can be taken.” [P1]*

### 3.3 Key points regarding implementation

#### 3.3.1 Actively involving community nurses

Almost all community nurses indicated that the need for change and the ‘win’ of the new practice needed to be clearly communicated to all those involved, especially to the community nurses to ensure normalization of the practice. Several community nurses explained that previous implementation processes of other innovations were not successful because workforces were set up without including the community nurses for input or collaboration:

*“Our opinions were simply not listened to. We found that very annoying. More colleagues came and said it didn't work. And the persons who set it up just didn't want to hear that. They said: no, you have to do this, you have to do that. But that's not how it works for us.”[P5]”*

Most community nurses agreed that the implementation would be an investment in time that could create friction. However, as one community nurse explained, clearly communicating what the advantages were and what it would yield for the community nurses might help in normalization of the new process:

*“It's important to communicate the benefits of a new approach. Sure, the process costs time, but if you can convey that message clearly and convincingly, the implementation process will go smoother.”[P17]*

They mentioned that it might help to assign some enthusiastic nurses with knowledge and experience in advance care planning who can be more closely involved during the whole implementation process and inspire other colleagues:

*“And you do not have to involve them all, but if you have two [community nurses] from each organization who communicate to the rest, you will be much more willing to cooperate.” [P11]*

#### 3.3.2 Need for a digital central health platform

All community nurses mentioned that a digital central health platform was an essential element to register and consult advance care planning agreements. They emphasized that this platform can make or break the successful implementation of the pathway. Crucial for many community nurses was a good workability of the system before implementation, as well as while visiting a client. On the same time, community nurses voiced their doubts based on their previous experiences on the development of digital systems between homecare organizations, general practitioners, and hospitals:

*“It's something that has been talked about for years with promises of implementation. *Will it take another 20 years to see it fully realized?” [P14]**

#### 3.3.3 Starting small

Most community nurses indicated the importance of starting implementation on a small scale to gain insight into facilitating and hindering factors and to prevent a large burden on the involved stakeholders; otherwise the initial implementation would not be successful. If successful, the initial small-scale implementation could help in providing success stories that can help in communicating the win for the broader implementation.

*“If you keep it on a smaller scale, you can filter out first before spreading it to a 100 people. Of course, the other 40 people may still have their own problems. But I believe that implementing it on a small group is always a good idea.” [P7]*

### 3.4 Evaluation of the new practice

#### 3.4.1 Follow-up of advance care planning

Community nurses indicated that registered wishes and preferences regarding advance care planning should be evaluated periodically with clients and relatives.

*“That follow-up of the [advance care planning] form. That is important. That it is not completed just once and then no longer necessary. Then you have to keep working on it.” [P19]*

Community nurses suggested that this should be done one or two times a year or when the medical or psychosocial condition of the client changes. They mentioned that the structured work process should describe which healthcare professional is responsible for the follow-up of advance care planning agreements. Furthermore, they suggested that an automatic pop-up in the central health system might help to remind healthcare professionals to evaluate advance care planning agreements.

#### 3.4.2 Acceptance and effectiveness

Community nurses mentioned several points that were important when evaluating the acceptance and effectiveness of the new practice. First of all, evaluation should include measures to assess both clients’ and healthcare providers’ satisfaction with the transmural care pathway.

*“Well, if everything, the care with the general practitioner and with us, has gone well, if the client and the family are satisfied. […] Then I think it was successful.” [P5]*

In addition, also the evaluation of experiences with the standardized documentation were mentioned. Community nurses expected an increased administrative burden due to the new standardized form. They elaborated that they were already using many forms for different purposes and that the advance care planning documentation form would be ‘yet another form’.

Next to the acceptance, also the effectiveness of the new format needed to be measured. Several community nurses indicated that of particular importance would be the minimal completion of essential sections; namely, the resuscitation status and the identification of a legal representative, including their contact information. Furthermore, community nurses also mentioned that the evaluation might consist of quantifying the extent to which the new format was utilized by determining the percentage of completed forms within a given time frame relative to the total number of clients, as elaborated on by this community nurse:

*“The number of completed forms. Because then you also know, there may be three forms completely filled out and we're doing it as desired, but if there are still 20 clients we're not doing anything with, then has it been effective?” [P8]*

## 4 Discussion

We examined the perspectives of community nurses on the feasibility of a transmural care pathway for advance care planning for older persons. Four themes were identified: (1) views on the transmural care pathway, (2) community nurses’ needs to fulfil their role, (3) key points regarding implementation and (4) evaluation of the new practice. In general, community nurses expected that the uniform work process and documentation form might improve the collaboration between all involved healthcare professionals and could contribute to timely palliative care for older persons. Based on community nurses perspectives’, several points of attention were identified that need to be considered before implementation of the transmural care pathway: (1) clear roles and responsibilities in the transmural care pathway, (2) standardized registration of advance care planning, and (3) close involvement of community nurses during implementation.|

### 4.1 Clear roles and responsibilities in the transmural care pathway

Community nurses emphasized that a structured pathway could be an important facilitator for the implementation of advance care planning within their region, as this is currently missing. Previous researchers also concluded that there was a lack of clear organisational policy and delineation of roles and responsibilities for advance care planning (Bolt et al., 2021; Poveda-Moral et al., 2021; Wilkin et al., 2024). The Rainbow Model of Integrated Care of Valentijn et al. (2015) acknowledges the complexity of integrating healthcare interventions in primary care (Valentijn et al., 2015). They described that the integration of interventions needed to take place on the clinical level, but also on the professional, organizational, and system levels. For successful implementation, actions on all levels are needed. Our transmural care pathway for advance care planning provides guidance for all involved healthcare professionals on the performance of advance care planning for older persons, their roles and responsibilities in the uniform work process, and the interprofessional collaboration between several organizations and care settings.

In the transmural care pathway, community nurses will have a leading role in the initiation and provision of interprofessional advance care planning. This differs from the current Dutch usual care, where nurses are not usually involved, and advance care planning is mainly the responsibility of general practitioners and medical specialists. However, advance care planning conversations are often initiated late in the disease trajectory and are mainly medically focussed (Dixon and Knapp, 2018). From previous research, nurses mentioned that they were able to identify patients with palliative care needs but felt that it was physicians’ responsibility. As a result, nurses felt reluctant to discuss their observations with physicians and to play a role in advance care planning (Flierman et al., 2019; van Doorne et al., 2023). Giving nurses responsibility to initiate advance care planning, in close collaboration with physicians, might facilitate timely palliative care for older persons. Furthermore, their expertise to look holistically at their clients’ needs might be a valuable addition to physicians’ expertise on discussing preferred treatment and treatment limitations (Dixon and Knapp, 2018). Community nurses can help to address (non-medical) life goals and wishes for (future) care that are important for older persons’ quality of life. This might contribute to a more holistic provision of palliative care. However, community nurses in our study mentioned that, before participating in the transmural care pathway, they were in need of additional education regarding advance care planning. Insufficient knowledge and conversational skills are known as important barriers in the provision of advance care planning (Blackwood et al., 2019; Wilkin et al., 2024). The Rainbow Model of Integrated Care describes that functional and cultural preconditions enable the integration of care, such as creating a learning culture (Valentijn et al., 2015). In this transmural care pathway, additional interprofessional training with healthcare professionals from varying care organizations and care settings should be provided before implementation.

One of the main characteristics of advance care planning is that it involves a process of shared-decision making between clients and healthcare professionals (Brinkman-Stoppelenburg et al., 2014; Rietjens et al., 2017). Community nurses indicated that clients could be more empowered within the new practice to ensure the feasibility of implementation. They suggested developing a specific client version of the advance care planning form and thus giving clients and relatives their own role in the pathway. Previous researchers have shown that shared decision making between healthcare professionals and patients regarding palliative care might facilitate implementation (Mc Broese et al., 2021). However, this is currently not provided within transmural care interventions (Kraun et al., 2023). The input of community nurses within this study has led to the development of a preparation tool for patients to get ready for a first conversation about advance care planning. The next step is that this form will be tested in practice, together with the documentation form for healthcare professionals.

### 4.2 Standardized registration of advance care planning

Community nurses mentioned that the standardized documentation form might provide guidance in advance care planning conversations and the standardized registration of older persons’ goals and wishes. They expected that this might help to access previously registered advance care planning agreements more easily. Community nurses indicated that the current lack of standardization between systems significantly impeded efficient collaboration and hand-overs, not only for advance care planning but also for providing efficient care in general.

In the literature, digital documentation, storage, and retrieval of advance care planning documents are often perceived as significant issues across systems of care (Bolt et al., 2021; Standing et al., 2020; Walker et al., 2018). To improve the feasibility of advance care planning interventions and palliative care for older persons, healthcare policy should be aimed at developing initiatives to enhance advance care planning information exchange between different digital systems. Within the transmural care pathway, a pilot to digitally register advance care planning agreements that are visible for all involved healthcare professionals, clients, and relatives will start in 2025. This pilot will provide important information regarding the barriers and facilitators of digital exchange of advance care planning information and might contribute to the feasibility of transmural palliative care.

### 4.3 Close involvement of community nurses during implementation

Community nurses mentioned the importance of being involved early in the implementation of the new transmural care pathway. They would like to have the opportunity to give their input and collaborate with other involved healthcare professionals on the proposed work process. Involving community nurses from the beginning might contribute to the feasibility of the pathway, as it might increase ownership and responsibility. Furthermore, assigning some community nurses as local champions might help to facilitate or promote the new uniform work process (Santos et al., 2022). The importance of champions in implementation of complex health interventions has been extensively elaborated upon in implementation literature (Schon, 1963; Warrick, 2009), also specifically in advance care planning (Francke et al., 2020). Within this transmural care pathway, local champions within homecare teams could be community nurses who have a special interest in advance care planning and often have an above-average understanding of advance care planning provision. They can be vital in making advance care planning processes more efficient, increase awareness and knowledge of advance care planning in colleagues, and facilitate implementation of new advance care planning initiatives. In addition, general practitioners or medical specialists involved in the transmural care pathway could be appropriate local champions as the pathway will be applied in several care settings.

## 5 Strengths and limitations

To the best of our knowledge, we are the first to examine the perspectives of community nurses on the feasibility of a new transmural care pathway for advance care planning. The Normalization Process Theory was used as a conceptual framework to provide in-depth insights into barriers and facilitators in the normalization of the new practice. This framework has been used previously in qualitative feasibility studies, and its constructs have been well-validated, ensuring validity in the current study (May et al., 2018; McEvoy et al., 2014).

Some limitations need to be considered. First, by including only community nurses’ perspectives, insights regarding the feasibility and implementation from other stakeholders, such as general practitioners, clients, and informal caregivers, were excluded. However, community nurses will have a central role in the new transmural care pathway and their perspectives resulted in a rich and detailed narrative on the feasibility of the new advance care planning practice. Furthermore, community nurses’ current role in advance care is often underused. We have provided valuable insights into their expertise and the important contribution they can have in a transmural care pathway. Second, using a purposeful sampling strategy may have resulted in selection bias, limiting the generalizability of the findings (Palinkas et al., 2015). However, the generalizability of the sample was guaranteed in other ways by including community nurses from all different homecare organizations allied to the initiative and from different locations. Third, data collection and analysis were mainly conducted by a single researcher, introducing the possibility of researcher bias. However, this concern was mitigated by developing and revising the topic guide in collaboration with multiple researchers (PJ, RE, MvR). Furthermore, repeated discussion rounds were held at every phase of the research, resulting in investigator triangulation. The next step is to perform a pilot study and test the transmural care pathway in practice. A comprehensive process evaluation during the pilot will provide more information of the feasibility of the pathway in practice.

## 6 Conclusions

We have highlighted important factors, from the perspectives of community nurses, that need to be considered in the implementation of a new transmural care pathway for advance care planning. A clear division of roles and responsibilities, standardized registration of advance care planning, and involvement of community nurses during the whole implementation process were mentioned as important enabling factors. This knowledge and timely involvement of community nurses might contribute to the successful implementation of a transmural care pathway that aims to enhance the quality of palliative care for older persons.

## Funding sources

This work was supported by the Netherlands Organisation for Health Research and Development (ZonMw) grant number 80-86300-98-118 to MvR. The sponsors had no role in study design, data collection and analysis, and neither in the preparation or publication of the manuscript.

## CRediT authorship contribution statement

**Patricia Jepma:** Writing – review & editing, Writing – original draft, Project administration, Methodology, Formal analysis, Data curation, Conceptualization. **Roel Eijk:** Writing – review & editing, Writing – original draft, Visualization, Validation, Project administration, Methodology, Formal analysis, Data curation, Conceptualization. **Annigje A.E. Bos:** Writing – review & editing. **Noor Toet:** Writing – review & editing, Resources, Project administration, Conceptualization. **Corine H.M. Latour:** Writing – review & editing, Supervision, Resources. **Bianca M. Buurman:** Writing – review & editing, Supervision, Resources, Conceptualization. **Marjon van Rijn:** Writing – review & editing, Supervision, Resources, Methodology, Funding acquisition, Formal analysis, Data curation, Conceptualization.

## Declaration of competing interest

The authors declare the following financial interests/personal relationships which may be considered as potential competing interests: Marjon van Rijn reports financial support was provided by Netherlands Organisation for Health Research and Development. If there are other authors, they declare that they have no known competing financial interests or personal relationships that could have appeared to influence the work reported in this paper.

## Appendix Supplementary materials

Image, application 1
