# Supplementary material for: Feasibility of a new transmural care pathway for advance care planning for older persons: A qualitative study into community care registered nurses’ perspectives
Source: Int J Nurs Stud Adv. 2024 Nov 8;7:100264. doi: 10.1016/j.ijnsa.2024.100264 (PMC11612358; doi:10.1016/j.ijnsa.2024.100264)
Supplement: Supplementary file 1 [file mmc1.docx]

**Supplementary file 1. Main constructs of the Normalization Process Theory**

| **Main constructs** | **Definitions** |
| --- | --- |
| Coherence | Coherence is defined as *“the sense-making work that people do individually and collectively when they are faced with the problem of operationalizing some set of practices”.^1^* It refers to determining and comprehending what people do when attempting to understand a new practice.^2^ |
| Cognitive participation | Cognitive participation is defined as *“the relational work that people do to build and sustain a community of practice around a new technology or complex intervention”.^1^* It refers to determining and comprehending what people do when contemplating and organising themselves and others to participate in a new practice.^2^ This also included the relational work that is done by actors to build a collective with shared agreement and engagement in the new practice. |
| Collective action | Collective action is defined as *“the operational work that people do to enact a set of practices”.^1^* It refers to determining and comprehending what people do when actually enacting a practice.^2^ |
| Reflexive monitoring | Reflexive monitoring is defined as *“the appraisal work that people do to assess and understand the ways that a new set of practices affect them and others around them.”^1^* It refers to determining and comprehending the formal and informal processes concerned with monitoring and evaluating the new practice.^2^ |

**References**

1. May C, Rapley T, Mair FS, Treweek S, Murray E, Ballini L, et al. Normalization Process Theory Online Users' Manual, Toolkit and NoMAD instrument. 2015.

* Definitions are cited from the coherence, cognitive participation, collective action and redlexive monitoring section of the online users' manual.

2. May C, Finch T. Implementing, Embedding, and Integrating Practices: An Outline of Normalization Process Theory I I. 2009.
